# “I Never Considered That”: The Impact of Engaging Older Adults in Research and Strategies for Making It Work

**DOI:** 10.1093/geroni/igaf122.137

**Published:** 2025-12-31

**Authors:** Erin McGaffigan, Marc Cohen, Sophia Webber, Melissa Destrampe, Myrna Finn, Janet Sasser, Linda Alloy

**Affiliations:** Collective Insight, North Reading, Massachusetts, United States; University of Massachusetts Boston, Boston, Massachusetts, United States; Collective Insight, North Reading, Massachusetts, United States; Collective Insight, North Reading, Massachusetts, United States; Osher Lifelong Learning Institute, Pittsfield, Massachusetts, United States; Los Angeles, California, United States; Brookline, Massachusetts, United States

## Abstract

Researchers often lack training and resources to meaningfully engage older adults in research. As a result, those most impacted by aging research- older adults- are often left out of the design and implementation. The Aging PCOR Learning Collaborative, a PCORI-funded project co-lead by Collective Insight and the LeadingAge LTSS Center@UMass Boston, encouraged researchers to engage older adults as partners so research agendas and scholarly activities better reflect age-inclusive perspectives. The Learning Collaborative engaged older adults and researchers to design educational tools and trainings to build researchers’ engagement capacity, reaching more than 800 aging-focused researchers, PhD students, and others in the aging research community to do just that. Among these activities was an Aging Research Advisory program, which provided opportunities for older adults to inform researchers and students on their aging-focused research activities. Ten older adults participated in introductory research training. They then hosted 8 advisory sessions from Fall 2023 to Summer 2024, providing feedback to 4 researchers on their research priorities and methods. In Spring 2025, Collective Insight and the LeadingAge LTSS Center@UMass Boston partnered with older adult research advisors to co-investigate the Aging Research Advisory program process and outcomes. In this presentation, we share our findings from this co-investigation, including the impact of advisory sessions on improving research relevance, design, and recruitment while also challenging researcher bias. We will also explore the specific strategies used to engage older adults as research advisors as well as co-researchers and co-writers when analyzing this model for older adult engagement across higher education.

